# Neuronal accumulation of unrepaired DNA in a novel specific chromatin domain: structural, molecular and transcriptional characterization

**DOI:** 10.1186/s40478-016-0312-9

**Published:** 2016-04-22

**Authors:** Jorge Mata-Garrido, Iñigo Casafont, Olga Tapia, Maria T. Berciano, Miguel Lafarga

**Affiliations:** Departamento de Anatomía y Biología Celular and “Centro de Investigación Biomédica en Red sobre Enfermedades Neurodegenerativas (CIBERNED)”, Universidad de Cantabria-IDIVAL, Avda. Cardenal Herrera Oria s/n, Santander, Spain

**Keywords:** DNA damage and repair centers, Accumulation of unrepaired DNA, Neurons, Nuclear compartments, Transcription, Genome stability

## Abstract

There is growing evidence that defective DNA repair in neurons with accumulation of DNA lesions and loss of genome integrity underlies aging and many neurodegenerative disorders. An important challenge is to understand how neurons can tolerate the accumulation of persistent DNA lesions without triggering the apoptotic pathway. Here we study the impact of the accumulation of unrepaired DNA on the chromatin architecture, kinetics of the DNA damage response and transcriptional activity in rat sensory ganglion neurons exposed to 1-to-3 doses of ionizing radiation (IR). In particular, we have characterized the structural, molecular and transcriptional compartmentalization of unrepaired DNA in persistent DNA damaged foci (PDDF). IR induced the formation of numerous transient foci, which repaired DNA within the 24 h post-IR, and a 1-to-3 PDDF. The latter concentrate DNA damage signaling and repair factors, including γH2AX, pATM, WRAP53 and 53BP1. The number and size of PDDF was dependent on the doses of IR administered. The proportion of neurons carrying PDDF decreased over time of post-IR, indicating that a slow DNA repair occurs in some foci. The fine structure of PDDF consisted of a loose network of unfolded 30 nm chromatin fiber intermediates, which may provide a structural scaffold accessible for DNA repair factors. Furthermore, the transcription assay demonstrated that PDDF are transcriptionally silent, although transcription occurred in flanking euchromatin. Therefore, the expression of γH2AX can be used as a reliable marker of gene silencing in DNA damaged neurons. Moreover, PDDF were located in repressive nuclear environments, preferentially in the perinucleolar domain where they were frequently associated with Cajal bodies or heterochromatin clumps forming a structural triad. We propose that the sequestration of unrepaired DNA in discrete PDDF and the transcriptional silencing can be essential to preserve genome stability and prevent the synthesis of aberrant mRNA and protein products encoded by damaged genes.

## Introduction

Cellular DNA damage response (DDR) is a molecular signaling pathway that is strongly induced by cytotoxic DNA lesions, such as double strand breaks (DSBs) which are produced by endogenous or exogenous genotoxic agents. In neurons, the DDR is mediated by the kinase ATM, which phosphorylates crucial protein partners in this pathway [1]. Mammalian neurons are highly vulnerable to DNA damage due to their high metabolic rate for energy production, generating cytotoxic reactive oxygen species (ROS) that can produce oxidative DNA damage. In addition, the relaxed chromatin configuration (euchromatin) of the majority of neuronal populations facilitates genotoxic agents gaining access to DNA and disrupting its structure [2, 3]. Cytotoxic DSBs can be derived from the conversion of single-strand breaks (SSBs) into DSBs or can be induced by environmental agents, such as ionizing radiation (IR), including X-rays, and chemotherapeutic drugs. They are especially detrimental for neurons as they affect genome integrity and global transcriptional activity [3–5]. Moreover, DSBs can produce energy starvation given that the DDR is a very high ATP consuming process [6]. Since post-mitotic neurons lack sister chromatids that serve as a template to ensure “error-free” repair by homologous recombination (HR), DSBs need to be repaired by non-homologous end joining (NHEJ) [7]. Due to the fact that DSB ends need to be processed before religation, errors can be introduced in NHEJ repair, resulting in neuronal dysfunction which ultimately contribute to neurodegeneration [8].

In addition to diseases with neurological manifestations caused by mutations in DNA repair factors, there is increasing evidence that defective DNA repair with an accumulation of DNA lesions and loss of genome stability underlies aging and many neurodegenerative disorders in human patients and animal experimental models [6, 9–12]. It has recently been reported that accumulated DNA damage can produce a deregulated DDR, leading to a senescence-like phenotype in neurons [9].

DDR occurs in the context of chromatin and requires elaborated epigenetic changes of histones in DNA damaged sites and flanking regions to stabilize broken DNA ends, in order to facilitate access of repair factors to damaged sites [8, 13]. Notwithstanding the extensive evidence of DNA damage-associated changes in the epigenome, it still remains unclear how neurons tolerate the accumulation of DNA lesions and how neurons process DNA damage in chromatin compartments and sequestrated unrepaired DNA in a few persistent DNA damage foci (PDDF) with a non-random spatial organization.

In a previous study, using an experimental model of DNA damage in rat sensory ganglion neurons (SGNs) with IR (4 Gy), we demonstrated that the neuronal DDR includes the formation of two categories of DNA-damage chromatin compartments [3]. The first are transient small and very numerous foci, which disappear within the first day post-IR, reflecting an effective DNA repair. The second consist of a few PDDF where unrepaired DNA is accumulated and remained at 15 days post-IR. Furthermore this neuronal DDR does not induce apoptosis but triggers G0-G1 cell cycle transition [3]. In this context, SGNs exposed to IR provide an excellent experimental system for investigating the nuclear organization and fate of unrepaired DNA accumulated in PDDF. Moreover, IR with a sub-lethal dose (4 Gy) in rodents triggers DDR, prevents neuronal apoptosis and makes it possible to study the long-term compartmentalization and dynamic of unrepaired DNA and its relationships with specific chromatin modifications and transcription rate. Finally, due to the absence of blood brain barrier in peripheral ganglia, the DNA damage-induced dysfunction of SGNs is a main component in peripheral neuropathies caused by cancer chemotherapy [14, 15]. In this study we analyze the following in irradiated SGNs i) the spatiotemporal organization of PDDF, ii) the molecular composition of these foci, iii) the ultrastructural compartmentalization of PDDF in cleared chromatin domains with unfolded chromatin fibers, iv) the transcriptional activity of PDDF and flanking chromatin domains, and v) the specific spatial association of PDDF with nuclear compartments which promotes gene silencing.

## Materials and methods

### Animals

Experiments were designed and performed to minimize the use of animals using a total of 72 young (30 days old) male Sprague-Dawley rats, distributed in a control (non-irradiated, *n* = 9) and five experimental groups treated with X-ray ionizing radiation (*n* = 9 per group). The animals were housed with a 12-h light/dark cycle and had free access to food and water. The animals were kept, handled, and sacrificed in accordance with the directives of the Council of the European Communities and current Spanish legislation, and the experiments were approved by the Bioethical Committee of the University of Cantabria.

### X-ray irradiation

Exogenous DNA damage was induced by X-Ray irradiation using an X-Ray generator system (Maxishot-d, Yxlon, Int. USA) equipped with an X-Ray tube which works at 200 kV and 4.5 mA. The animals, deeply anesthetized with pentobarbital (50 mg/kg), were placed 25 cm away from the X-Ray source that generated an X-Ray beam with an absorbed dose rate of approximately 0.9 Gy/min. The animal’s body was protected with a lead tube, exposing only the head, and the beam focused on the head to avoid adverse effects on the bone marrow, spinal cord and any other tissues produced by global animal radiation. The animals were exposed to IR for 4 min and 20 s in order to administrate a sub-lethal dose of 4Gy, a reference dose in DNA damage/repair experiments [3]. For this work we used control and irradiated animals with one, two or three doses of IR (4Gy each) as indicated in the experimental plan of the Table 1. The animals were sacrificed and the trigeminal sensory ganglia were processed for different cell biology and biochemical methods.

**Table 1:** Summary of the experimental plan

Animals	30 Days old	45 Days old	60 Days old	75 Days old
Control	-	Sacrifice	-	-
1 Dose of X-Ray irradiation	Irradiation 4Gy	Sacrifice	-	-
	Irradiation 4Gy	-	Sacrifice	-
	Irradiation 4Gy	-	-	Sacrifice
2 Doses of X-Ray irradiation	Irradiation 4Gy	Irradiation 4Gy	Sacrifice	-
3 Doses of X-Ray irradiation	Irradiation 4Gy	Irradiation 4Gy	Irradiation 4Gy	Sacrifice

### Immunofluorescence and confocal microscopy

For light immunocytochemistry, the animals (*n* = 3 animals per group) deeply anesthetized as described above were perfused with the fixative solution containing 3.7 % formaldehyde (freshly prepared from paraformaldehyde) in PBS. Tissue fragments of trigeminal ganglia were removed and washed in PBS. For immunofluorescence, each tissue fragment was transferred to a drop of PBS on a siliconized slide (SuperFrostPlus, Menzel-Gläser, Germany) and squash preparations of dissociated neurons were performed following the previously reported procedure [16]. The samples were sequentially treated with 0.1 M glycine in PBS for 15 min, 3 % BSA in PBS for 30 min and 0.5 % Triton X-100 in PBS for 45 min. They were then incubated with the primary antibody overnight at 4 °C, washed with 0.05 % Tween 20 in PBS, incubated for 45 min in the specific secondary antibody conjugated with FITC or TexasRed (Jackson, USA), washed in PBS and mounted with the antifading medium ProLong (Invitrogen, USA). Some samples were counterstained with DAPI, a cytochemical marker of DNA.

Confocal images were obtained with a LSM510 (Zeiss, Germany) laser scanning microscope and using a 63x oil (1.4 NA) objective. In order to avoid overlapping signals, images were obtained by sequential excitation at 355, 488 and 543 nm in order to detect DAPI, FITC and Texas Red, respectively. Emission signals were detected at 405–450 nm for DAPI, 505–530 nm for FITC and >560 for Cy3 or Texas Red. Images were processed using Photoshop software.

The proportion of damaged SGNs containing IR-induced PDDF and the number of foci per neuron was determined by direct examination of dissociated neurons, in which the whole neuronal body is preserved, immunostained for γH2AX and using a 40X objective. At least 100 neurons per animal were examined (*n* = 3 animals per group). Planimetric measurements of PDDF areas were made on confocal microscopy images of sensory ganglion neurons immunostained for the γH2AX, using a 63 X (1.4NA) immersion oil objective. Image processing and measurement steps were performed on ImageJ, public domain software for image analysis (NIH, Bethesda, Maryland, USA; http://rsb.info.nih.gov/ij/). The average PDDF area was estimated on at least 30 nuclear confocal sections of neurons per animal (*n* = 3 animals per group). Average values were pooled for subsequent graphing and analysis. Data were analyzed using Microsoft Excel and the analysis of variance was used to determine the statistical significance of differences between control and irradiated neurons of sensory ganglia. Values are Means ± SD.

### Transmission electron microscopy

For conventional, immunogold and ultrastructural electron microscopy examination of SGNs, control and irradiated rats (*n* = 3 animals per group) were perfused under deep anesthesia with 3.7 % paraformaldehyde in 0.1 M cacodylate buffer for 10 min at room temperature. Small tissue fragments of trigeminal ganglia were washed in 0.1 M cacodylate buffer, dehydrated in increasing concentrations of methanol at −20 °C, embedded in Lowicryl K4 M at −20 °C and polymerized with ultraviolet irradiation. Ultrathin sections were mounted on nickel grids and were stained with lead citrate and uranyl acetate and examined with a JEOL 1011 electron microscope. Some ultrathin sections were processed for the EDTA staining procedure for ribonucleoproteins. For immunogold electron microscopy, sections were sequentially incubated with 0.1 M glycine in PBS for 15 min, 5 % BSA in PBS for 30 min and the primary antibody for 2 h at 37 °C. After washing, the sections were incubated with the specific secondary antibodies coupled to 10 nm gold particles (BioCell, UK; diluted 1:50 in PBS containing 1 % BSA). Following immunogold labeling, the grids were stained with lead citrate and uranyl acetate. As controls, ultrathin sections were treated as described above but with the primary antibody omitted.

### Run-on transcription assays in situ

Active transcription sites were labeled by the incorporation of 5’-fluorouridine (5’-FU) into nascent RNA. Briefly, under anesthesia both control and irradiated rats (*n* = 3 animals per group) were given an intravenous injection of 5’-FU (Sigma, UK) of a stock solution of 0.4 M 5’-FU in 0.9 % saline at doses of 5 μl/g. All animals were sacrificed after 45 min post-injection of the halogenated nucleotide and fixed by perfusion with 3.7 % paraformaldehyde in HPEM buffer (2x HPEM: Hepes, 60 mM; Pipes, 130 mM; EGTA, 20 mM; and MgCl_2_ · 6H_2_O, 4 mM) containing 0.5 % Triton X-100 for 10 min. Trigeminal ganglia were removed, washed in HPEM buffer containing 0.5 % Triton X-100 for 10 min and cut into small fragments. Then tissue fragments were washed in 0.1 M HPEM buffer, dehydrated in increasing concentrations of methanol at −20 °C, embedded in Lowicryl K4 M at −20 °C and polymerized with ultraviolet irradiation. Ultrathin sections were mounted on nickel grids and sequentially incubated with 0.1 M glycine in PBS for 15 min, 5 % BSA in PBS for 30 min and the mouse monoclonal anti-BrdU (clone BU-33, Sigma, UK) antibody (diluted 1:25 in 50 mM Tris–HCl, pH 7.6, containing 1 % BSA and 0.1 M glycine) for 1 h at 37 °C. After washing, the sections were incubated with an anti-mouse secondary antibody coupled to 15 nm gold particles (BioCell, UK; diluted 1:50 in PBS containing 1 % BSA). Following immunogold labeling, the grids were stained with lead citrate and uranyl acetate and examined with a JEOL 1011 electron microscope. As controls, ultrathin sections were treated as described above but with the primary antibody omitted.

### SDS-PAGE and Immunoblotting

Trigeminal ganglia from control and irradiated rats (*n* = 3 animals per group) were lysed using a Polytron PT-2000 (Kinematica®, Luzern-Switzerland) on ice in cold extraction buffer NETN [20 mM Tris–HCl pH 8.0, 500 mM NaCl, 1 mM EDTA] containing Benzonase (1 μL/1 mL lysis buffer) (Novagen) and supplemented with protease and phosphatase inhibitor cocktail (Halt™ Protease and Phosphatase inhibitor single use cocktail, Thermo Scientific, USA) and incubated for 30 min on ice. After centrifugation (12 min at 12000 rpm) at 4 °C the supernatant was frozen. Proteins were separated on SDS-PAGE gels and transferred to nitrocellulose membranes by standard procedures. Protein bands were detected with an OdysseyTM Infrared-Imaging System (Li-Cor Biosciences) according to OdysseyTM Western-Blotting Protocol. Immunoblots were developed with anti-mouse IRDye800DX or anti-rabbit IRDye700DX (Rockland Immunochemicals, USA) secondary antibodies.

### Antibodies

The primary antibodies used and their dilutions for immunofluorescence, immunogold electron microscopy and Western blotting are described in Table 2. Specific secondary antibodies conjugated with FITC, TexasRed or Cy3 (Jackson Lab., USA) were used for immunofluoescence.

**Table 2:** Antibodies used in this study

Antibody	Marker	Type	Origin (Reference)	Technique and Dilution
Anti-Alpha-Tubulin	Loading Control	Mouse Monoclonal	Sigma (T9026)	WB (1:1000)
Anti-BrdU	Transcription Assay	Mouse Monoclonal	Sigma (B8434)	IE (1:50)
Anti-Fibrillarin	Nucleolus	Mouse Monoclonal	ABCAM (ab4566)	IF (1:500)
Anti-Histone H2AX phospho-Ser139	DNA Damage	Mouse Monoclonal	Millipore (05–636)	WB (1:1000)
				IF (1:200)
Anti-HP1 gamma	Heterochromatin	Mouse Monoclonal	Millipore (05–689)	IF (1:100)
Anti-RNA Pol II H5 antibody	Transcription sites	Mouse Monoclonal	Covance (MMS-129R)	IE (1:50)
Anti-TMG Cap	Nuclear Speckle (IGC)	Mouse Monoclonal	Oncogene (NA02A)	IF (1:100)
Anti-WRAP53	DNA Damage	Mouse Monoclonal	ABNOVA (H00055135-M04)	WB (1:2000)
				IF (1:200)
				IE (1:100)
Anti-53BP1	DNA Damage/repair	Rabbit Polyclonal	Bethyl Laboratories (A300-272A)	WB (1:1000)
				IF (1:250)
				IE (1:50)
Anti-ATM Phospho-Ser1981	DNA Damage	Rabbit Polyclonal	Cell Signaling (4526)	WB (1:500)
				IF (1:100)
Anti-Coilin 210.4 antibody	Cajal Body	Rabbit Polyclonal	Provided by Prof. A.I. Lamond	IF (1:250)
				IE (1:50)
Anti-Fibrillarin 12.3 antibody	Nucleolus	Rabbit Polyclonal	Provided by Prof. M.Carmo-Fonseca	IE (1:100)
Anti-Histone H2AX phospho-Ser139	DNA Damage	Rabbit Polyclonal	Novus (NB100-384)	IF (1:200)
Anti-Trimethyl-Histone H4 (Lys 20)	Heterochromatin	Rabbit Polyclonal	Millipore (07–463)	IF (1:250)
Anti-Ubiquityl H2A	DNA Damage	Rabbit Polyclonal	Millipore (05–678)	WB (1:1000)
				IF (1:100)

*WB* western blotting, *IF* immunofluorescence microscopy, *IE* immunogold electron microscopy

## Results

### Organization and dynamics of PDDF induced by IR in SGNs

**Fig. 1:**
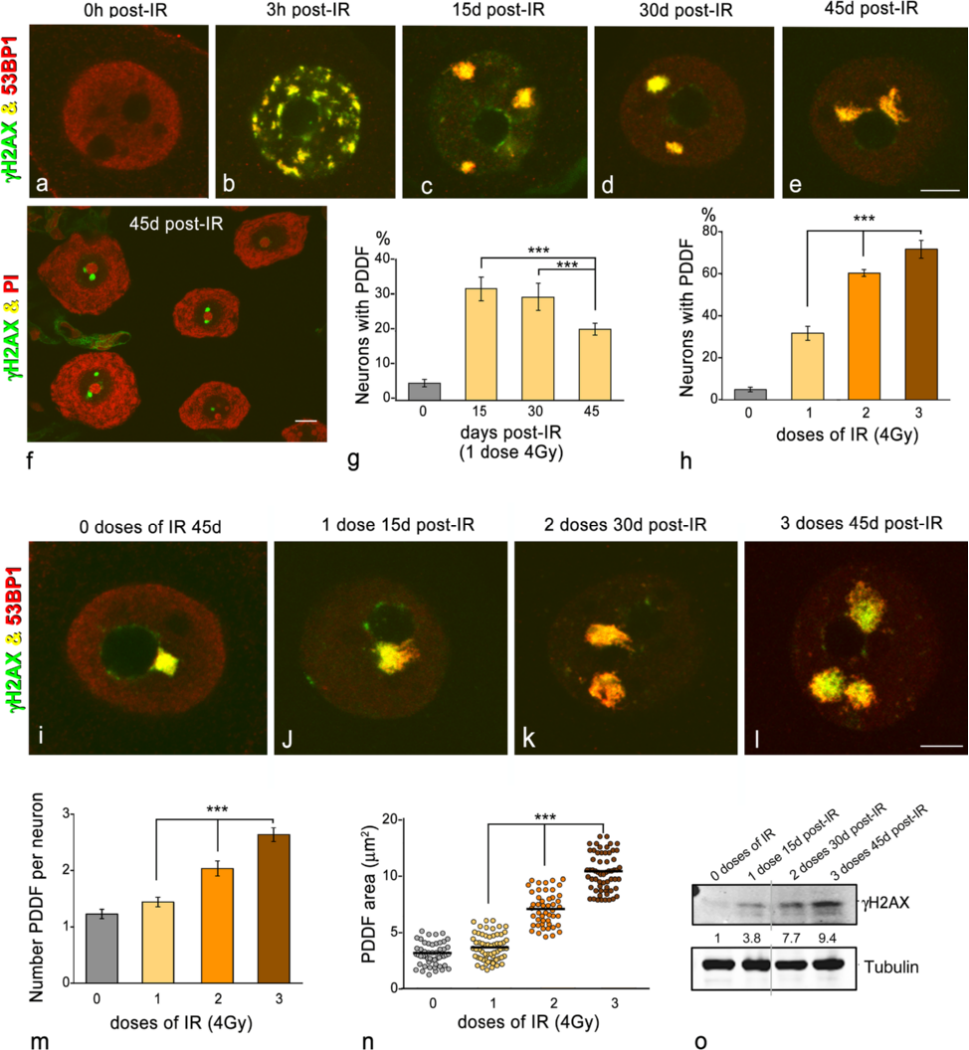
**a–e** Representative examples of double immunolabeling for γH2AX and 53BP1 in dissociated sensory ganglion neurons from control (**a**) and irradiated neurons at 3 h, 15d, 30d and 45d pos-IR (**b**–**e**). **a** Control neurons lack of γH2AX signal and exhibit a diffuse nucleoplasmic labeling for 53BP1. **b** At 3 h post-IR, γH2AX and 53BP1 colocalize in numerous DNA damage foci of variable size and distributed throughout the nucleus, excepting the nucleolus. **c–e** At 15d, 30d and 45d post-IR, one to three large PDDF appear intensely immunostained for γH2AX and 53BP1. Scale bar: 5 μm. **f** SGN perikarya immunolabeled for γH2AX and counterstained with propidium iodide (PI) illustrate the preferentially perinucleolar location of PDDF. Scale bar: 10 μm. **g** Proportion of SGNs containing γH2AX-positive PDDF at 15d, 30d and 45d post-IR, and irradiated with a single dose (4Gy) (data are mean ± SD from three independent experiments, at least 100 neurons per group were counted; ***p < 0.001). **h** Proportion of SGNs containing γH2AX-positive PDDF following the administration of one, two and three doses of IR, and 15 days after the last treatment. (data are mean ± SD from three independent experiments, at least 100 neurons per group were counted; ***p < 0.001). **i–l** Double immunolabeling for γH2AX and 53BP1 in SGNs from control (**i**) and irradiated neurons with one, two or three doses (**j–l**). Note a spontaneous perinucleolar PDDF in a control neuron and the dose-dependent increase of PDDF in irradiated neurons. Scale bar: 5 μm. (**m**, **n**) Mean number per neuron and size of PDDF following the administration of one to three doses of IR. Note the dose-dependent increase in the number and size of foci (data are mean ± SD from three independent experiments; ***p < 0.001). **o** Western blot analysis of phosphorylated histone H2AX at Ser 139 (γH2AX) in sensory ganglion lysates from controls and irradiated animals (*n* = 3 animals per group). The expression of γH2AX was induced with IR and its protein levels increased in dose-dependent manner. The expression of alpha-tubulin band was used as a protein loading control, and the fold increase estimated

The organization and dynamics of PDDF were analyzed in mechanically dissociated perikarya of SGNs exposed to one (4Gy), two (4Gy x 2) or three (4Gy x 3) doses of IR and double immunolabeled for phosphorylated histone H2AX (γΗ2ΑX), a well-established marker of DSBs [17, 18], and also 53BP1, a key factor that protects DNA ends from resection and promotes DNA repair by the NHEJ [19, 20]. In control neurons the nuclear expression of γΗ2ΑX was barely detectable, whereas 53BP1 displayed a diffuse nuclear expression (Fig. 1a). In contrast, numerous and small transient DNA damage foci immunoreactive for γΗ2ΑX and 53BP1 were observed at 3 h post-IR (Fig. 1b). As previously reported [3], most of them disappeared within the first 24 h post-IR indicating an effective DNA repair.

In this study we have focused on the organization and behavior of γΗ2ΑX- and 53BP1-positive PDDF that remained over longer terms, at 15, 30 and 45 days post-IR (Fig. 1c–f). They reflect the neuronal processing of irreparable or very slowly repaired DNA. A very small proportion of control neurons showed isolated PDDF, which presumably correspond to endogenous, spontaneous DNA damage (Fig. 1g, h). The global proportion of PDDF-containing SGNs after a single dose of IR was approximately 32 % at 15 days post-IR, decreasing significantly (20 %) at 45 days post-IR (Fig. 1g). These findings support slow, long-term DNA repair in this neuronal population. Furthermore, the proportion of neurons with PDDF significantly increased when SGNs were exposed to double or triple IR doses (up to 72 %), as compared with those irradiated with a single dose (Fig. 1h). This observation clearly indicates that the neuronal accumulation of unrepaired DNA in PDDF is dependent on the total dose of IR. Next we determined the mean number of PDDF per nucleus and the size of these foci in DNA damaged neurons. Whereas most of the neurons exposed to a single dose of IR carried one or less frequently two large PDDF at 15 days post-IR, the administration of two or three doses of IR was associated with a parallel significant increase in both the number and size of PDDF (Fig. 1i–l, graphs m, n), indicating a dose-dependent accumulation of DNA damage. These findings were confirmed by Western blotting for γΗ2ΑX in sensory ganglion lysates. While the phosphorylated H2AX was barely detectable in non-irradiated ganglion, a notable and dose-dependent increase of protein levels was observed in irradiated ganglion (Fig. 1o).

### PDDF concentrate essential components of the DNA damage/repair signaling pathway

**Fig. 2:**
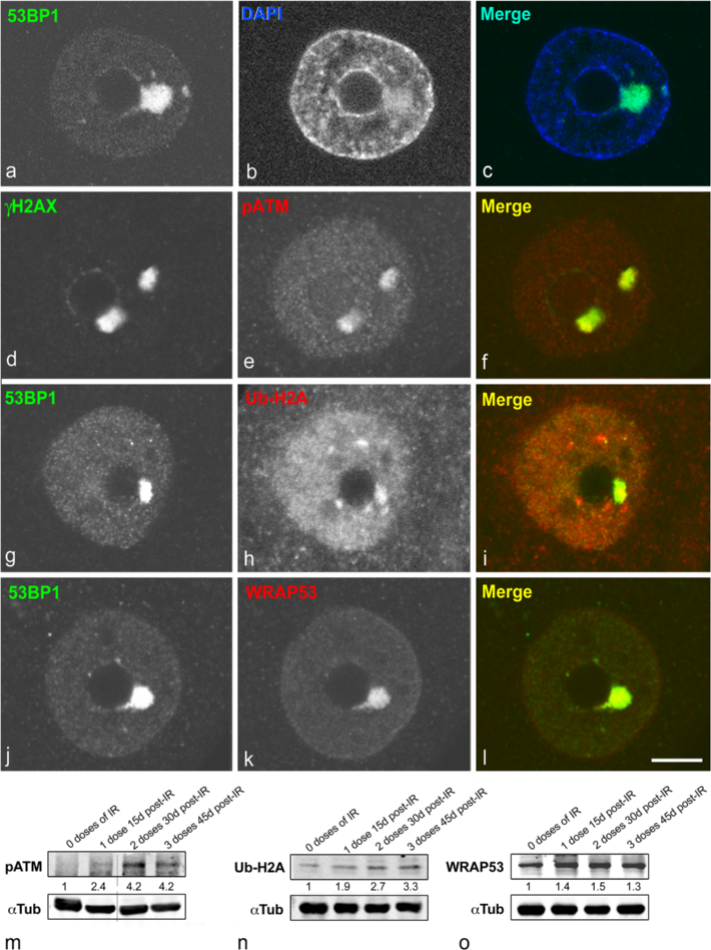
**a–c** Immunostaining of SGNs nuclei for 53BP1 in combination with DAPI demonstrated the presence of DNA in a PDDF. 45d post-IR. **d–l** Double immunolabeling for yH2AX in combination with pATM (**d–f**)**,** and 53BP1 in combination with either Ub-H2A (**g**–**i**) or WRAP53 (**j–l**) demonstrated the colocalization of all these DNA damage signaling and repair factors in PDDF. 45d post-IR. Scale bar: a-l = 5 μm. **m–o** Western blot analysis of the expression levels of pATM (**m**), Ub-H2A (**n**) and WRAP53 (**o**) in sensory ganglion of non-irradiated and irradiated rats exposed to one, two or thee doses of IR (4Gy each; *n* = 3 animals per group). Note the induction of pATM after IR, and the dose-dependent substantial increase of both pATM and Ub-H2A protein levels. A moderate increase over the basal levels in control ganglion of WRAP53 was also observed upon IR. The expression of alpha-tubulin band was used as a protein loading control, and the fold increase estimated

In order to rule out the possibility that PDDF were a simple DNA-free reservoir of protein components of DDR, we performed DNA staining with DAPI in combination with immunolabeling for γΗ2ΑX. As shown in Fig. 2a–c, DNA is present in PDDF, appearing as a diffuse signal of moderate intensity as compared with the strongly stained perinuclear heterochromatin.

In addition to γΗ2ΑX and 53BP1, we investigated the recruitment in PDDF of three essential factors for DNA repair: phosphorylated ATM (pATM), ubiquitylated H2A (Ub-H2A) and WRAP53 (WD40 encoding RNA antisense to p53) (*n* = 30 neurons per animal). Since ATM kinase is a fundamental DSBs sensor for triggering DDR [21], we analyzed the recruitment of active autophosphorylated ATM to PDDF. Double immunolabeling for pATM and γΗ2ΑX revealed the colocalization of both molecular components in the PDDF examined (Fig. 2d–f). Given that chromatin remodeling at DNA damage sites is required for repair factors can access to DSBs, we analyzed the ubiquitylation of the histone H2A and the recruitment of WRAP53 to PDDF. Whereas Ub-H2A promotes chromatin remodeling and the assembly of repair factors to DSBs [22, 23], WRAP53 regulates histone ubiquitylation and provides a scaffold for DNA repair factors [24]. Co-immunostaining of Ub-H2A or WRAP53 with 53BP1 revealed the concentration of the first two in 53BP1-positive PDDF, regardless of the IR dose that was administered (Fig. 2g–l). The IR-induced and dose-dependent increase in the expression levels of pATM and Ub-H2A was corroborated with Western blotting of sensory ganglion lysates (Fig. 2m, n). On the other hand, basal levels of WRAP53 were detected in control sensory ganglion and a slight increase in protein levels was registered after IR treatment (Fig. 2o). Collectively, the presence in PDDF of essential components of the DNA damage/repair machinery indicates that DNA damage signaling continues long-term after IR.

### Ultrastructural organization of chromatin in PDDF and flanking domains

**Fig. 3:**
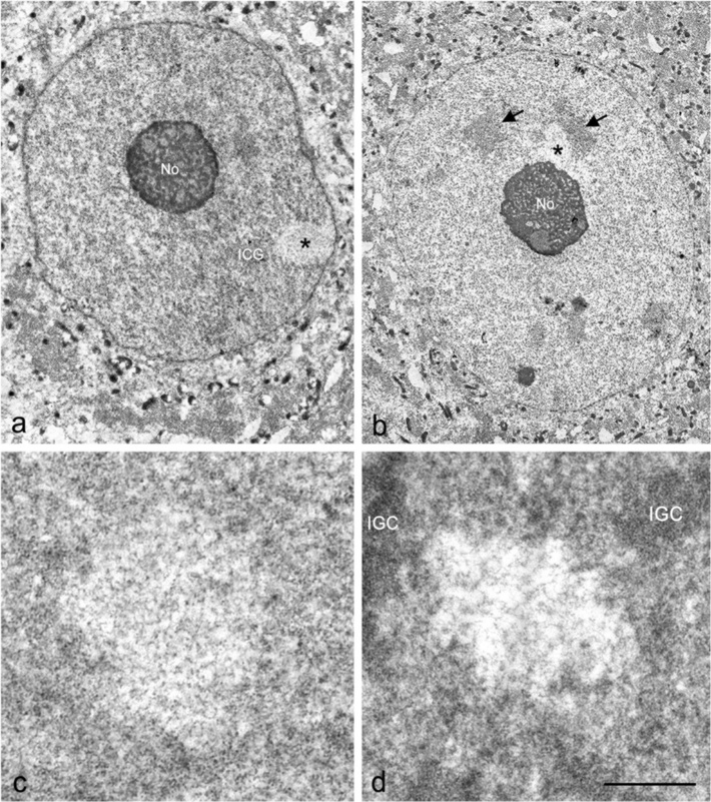
Electron micrographs of SGN nuclei illustrating the structure and organization of PDDF. 45d post-IR. **a**, **b** PDDF (asterisks) appeared as cleared areas in the euchromatin landscape associated with the nuclear envelope or with the nucleolus (No). Interchromatin granule clusters (arrows). Scale bar: 5 μm. **c**, **d** Detailed fine structure of a PDDF composed of a loosen network of chromatin fibers surrounded by euchromatin. Scale bar: 1 μm. Cytochemical staining for ribonucleoproteins revealed the lack of weak staining of a PDDF, whereas the adjacent euchromatin and, particularly, the interchromatin granule clusters (IGC) appeared intensely contrasted. Scale bar: 1 μm

To determine the structural chromatin organization in PDDF we performed conventional electron microscopy and ultrastructural immunogold for 53BP1 and WRAP53. By electron microscopy of tissue samples which were fixed with 4 % paraformaldehyde and embedded in Lowicryl 4KM, PDDF appeared as well-defined cleared nucleoplasmic areas in euchromatin regions and were preferentially located at the nucleolar and nuclear periphery (Fig. 3a, b). At a high magnification, PDDF exhibited deep relaxation of chromatin structure, which was composed of a network of tiny fibers and commonly surrounded by euchromatin regions (Fig. 3c). With the EDTA preferential staining for ribonucleoproteins [25], the RNA-rich nuclear structures, including the ribonuceoproteins of euchromatin and interchromatin granule clusters (“nuclear speckles” at light microscopy level, [26]), were intensely stained. In contrast, PDDF stood out against the adjacent euchromatin because of their electron-lucent appearance, indicating a reduced concentration of ribonucleoproteins (Fig. 3d).

**Fig. 4:**
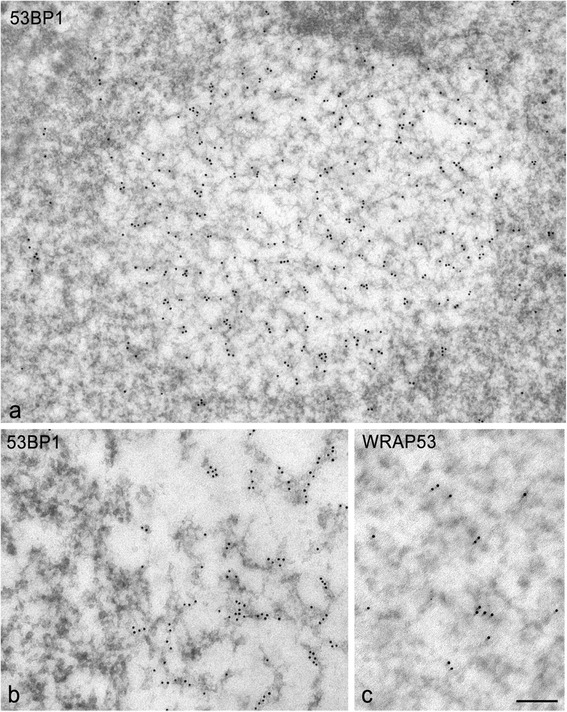
Immunogold electron microscopy for the detection of 53BP1 (**a**, **b**) and WRAP53 (**c**) in PDDF. 45d post-IR. Immunogold particles of 53BP1 immunoreactivity strongly decorated the network of chromatin fibers throughout the focus. **c** Similarly, immunogold labeling for WRAP53 was also detected on the fibers. Scale bar: *a* = 175 nm, *b* = 150 nm, *c* = 125 nm

With the immunogold electron microscopy for DNA repair factor 53BP1, PDDF were sharply defined by their intense immunolabeling (Fig. 4a). In particular, gold particles of 53BP1 immunoreactivity specifically decorated a fibers network within foci, whereas the associated nucleoplasm was free of labeling (Fig. 4b). Importantly, 53BP1-positive fibers ranged in diameter from smaller (about 11 nm), which may correspond to nucleosomal chains, to larger fibers (about 30 nm), a diameter characteristic of chromatin fibers [27]. This finding strongly supports the fact that the PDDF contain unfolded chromatin fibers with increased accessibility of DNA to repair factors. Regarding the immunogold labeling for WRAP53, gold particles also decorated PDDF fibers, although at a lower intensity than 53BP1 (Fig. 4c). No differences in the fine structure and immunogold labeling of PDDF were observed regardless of the total IR doses administered.

### PDDF are transcriptionally silent, but active transcription occurs in flanking chromatin

**Fig. 5:**
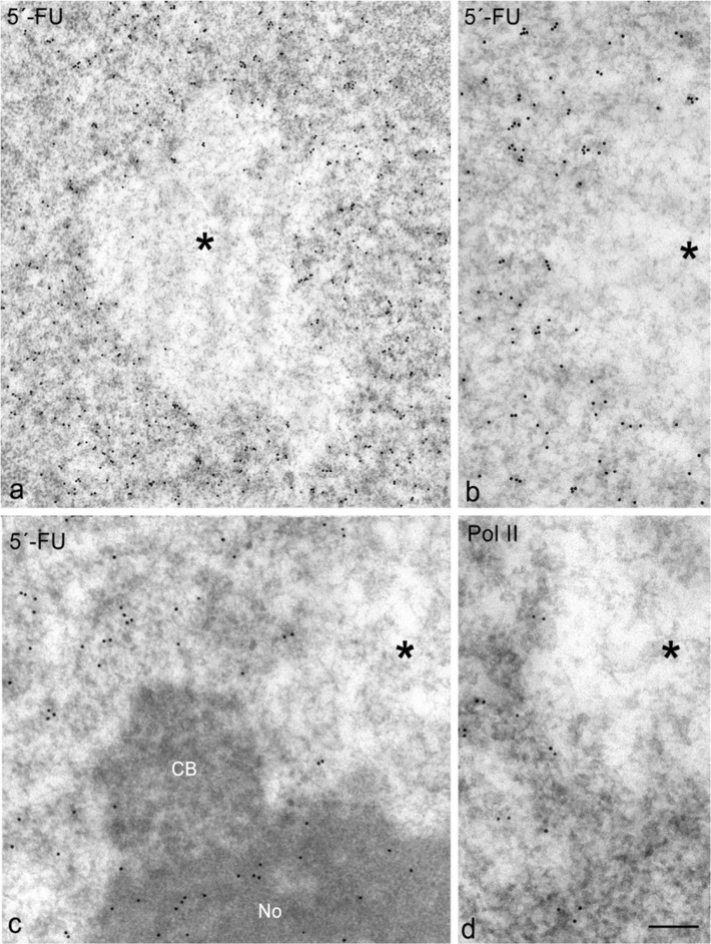
Immunogold electron microscopy transcription assay based on the incorporation of 5’-FU into nascent RNA after 45 min of the intraperitoneal administration of 5’-FU. 45d post-IR. **a** This PDDF (asterisk) lack of immunogold labeling indicating the absence of transcription. However, the euchromatin, including de domains flanking the PDDF appeared intensely labeled with gold particles of 5’-FU incorporation. Scale bar: 300 nm. **b** Detailed of 5’-FU incorporation in the euchromatin boundary of a PDDF (asterisk). Scale bar: 200 nm. **c** Detail of a PDDF (asterisk) associated with both the nucleolus (No) and a Cajal Body (CB). Note the absence of nascent RNA in the PDDF and in the transcription-free CB, whereas the nucleolus and adjacent euchromatin appeared immunogold labeled. **d** Immunogold electron microscopy for the active RNA polymerase II shows the absence of labeling in a PDDF (asterisk), whereas the surrounding euchromatin appeared decorated with gold particles Scale bar: 150 nm

To analyze the transcriptional activity in PDDF and flanking chromatin domains, we performed an *in situ* transcription assay using the high resolution immunogold electron microscopy. This procedure is based on the incorporation of the RNA precursor 5’-FU into nascent RNA. After a 45-min pulse of 5’-FU incorporation, immunogold particles decorated the extensive nuclear domains of euchromatin (Fig. 5a, b), where active protein-coding genes are distributed. Additionally, an intense 5’-FU incorporation was also detected in the dense fibrillar component of the nucleolus (Fig. 5c), the site of transcription of ribosomal genes [28]. As expected, immunogold particles were absent from transcription-free nuclear compartments such as Cajal bodies (Fig. 5c). Importantly, nascent RNA was not detected in PDDF, with their network of unfolded chromatin fibers appearing free of gold particles (Fig. 5a–c). However, an active transcription was observed in flanking euchromatin, establishing a sharply defined boundary between transcriptionally permissive chromatin and non-permissive DNA-damaged chromatin (Fig. 5a, b).

To further confirm that PDDF are transcriptionally silent, we performed immunogold electron microscopy for detecting RNA polymerase II (pol II), the enzyme that directs the transcription of protein-coding genes [29]. We used the antibody H5 which recognizes the active RNA pol II, hyperphosphorylated on Ser2, which is involved in the elongation phase of transcription [29, 30]. In accordance with the 5’-FU transcription assay, immunogold particles were absent from PDDF, although they decorated flanking euchromatin and other euchromatin regions (Fig. 5d).

### Spatial organization of PDDF and their associations with nuclear compartments

**Fig. 6:**
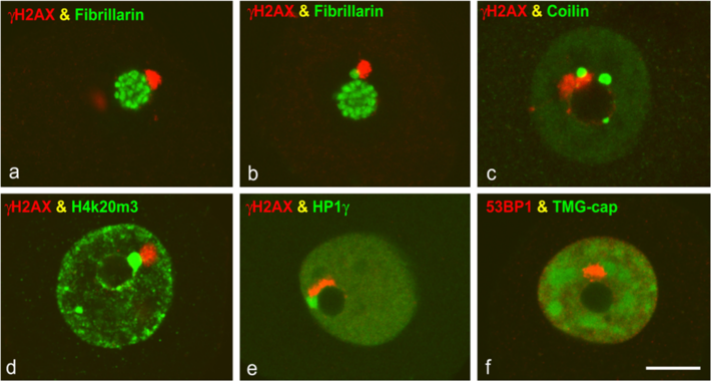
**a–e** Confocal microscopy images of SGN nuclei co-stained for γH2AX in combination with fibrillarin (**a**, **b**), coilin (**c**), histone H4K20me3 (**d**) and HP1γ (**e**). They illustrate the direct association of PDDF with a triad of structures: the nucleolus (**a**, **c**, **e**), Cajal bodies, immunolabeled for fibrillarin (**b**) or coilin (**c**), and perinucleolar heterochromatin clumps immunolabled for the tri-methylated histone H4K20me3 (**d**) or HP1γ (**e**). **f** Double immunolabeling for 53BP1 and TMG-cap illustrate the distribution of the nuclear speckles of splicing factors labelled with the anti-TMG-cap antibody and the close association of a PDDF with a speckle in the vicinity of the nucleolus. 45d post-IR. Scale bar = 5 μm

**Fig. 7:**
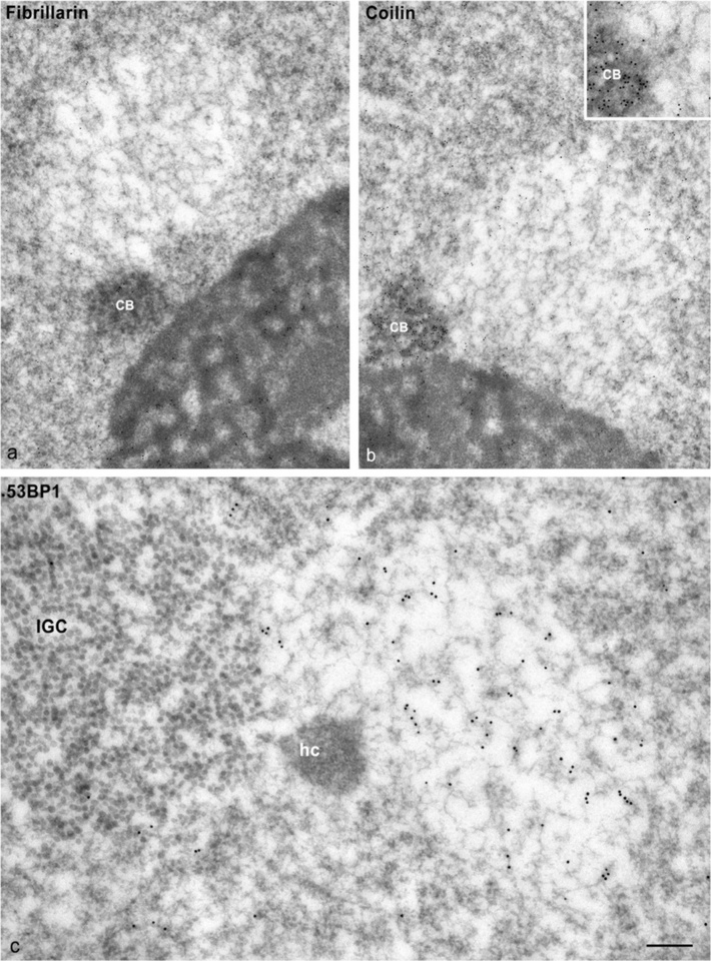
**a**, **b** Immunogold electron microscopy illustrating the direct association of PDDF with the nucleolus and a Cajal body labeled with the anti-fibrillarin antibody (**a**), and the nucleolus and a coilin-positive Cajal body (**b**). **c** A 53BP1-positive PDDF appears associated with a cluster of interchromatin granules (IGC) and a heterochromatin mass (hc). Scale bar: *a* = 500 nm, *b* = 400 nm and *c* = 100 nm

PDDF appeared in euchromatin domains, but they were apparently not randomly distributed within the neuronal nucleus. The quantitative analysis in SGNs immunolabeled for γH2AX revealed that approximately 70 % of PDDF were perinucleolar, whereas 20 % were distributed at the nuclear periphery and the remainder was located in other nuclear regions (Fig. 1f). Double immunolabeling for fibrillarin, a nucleolar marker [28], and 53BP1 confirmed the preferential spatial association of PDDF with the nucleolus (Fig. 6a). In this perinucleolar localization, PDDF frequently associated with Cajal bodies immunolabeled for fibrillarin (Fig. 6b) or coilin (Fig. 6c), a specific Cajal body marker [31–33]. Furthermore, a close association between PDDF and heterochromatin clumps, immunolabeled for the histone H4K20me3 or HP1γ (heterochromatin protein 1γ), was also observed at both perinucleolar and peripheral domains (Fig. 6d, e). Thus PDDF established spatial associations, but not co-localization, with a triad of nuclear structures: nucleolus, Cajal bodies and heterochromatin clumps. Occasional associations between PDDF and nuclear speckles immunostained with the anti-TMG-cap antibody, which recognizes the 5’ end of spliceosomal snRNAs [26], were detected (Fig. 6f). Immunogold electron microscopy with anti-fibrillarin and anti-coilin antibodies confirmed the association of PDDF with both Cajal bodies and nucleolus at the perinucleolar compartment (Fig. 7a, b). Similarly, the association of PDFF with both heterochromatin clumps and interchromatin granule clusters was validated with immunogold electron microscopy with the anti-53BP1 antibody (Fig. 7c).

## Discussion

Our results in healthy SGNs exposed to sub-lethal doses of IR for inducing DSBs demonstrate that unrepaired DNA is retained for extended periods of time, up to 45 days in our study, in a few PDDF. The number and size of these foci is dependent on the total dose of IR, indicating a direct relationship between DNA damage accumulation and PDDF formation. PDDF are not simply static DNA damage “scars” of the genome, but in fact dynamic and specifically compartmentalized chromatin domains that recruit essential factors for signaling and repair DNA, such as γH2AX, pATM, Ub-H2A and WRAP53. Furthermore, the progressive reduction of SGNs carrying PDDF over time of post-IR reported here supports that a slower DNA repair is still ongoing in some foci. This is consistent with the permanent enrichment of DNA damage signaling and repair factors in PDDF, independently of the post-IR time and the total dose of IR administered. Our study provides the first analysis on the organization and dynamics of persistent foci of damaged DNA in neurons.

Regarding the possible pathophysiological implications of the accumulation of unrepaired DNA damage in neuronal PDDF, the loss of genomic integrity may contribute to both cognitive ageing and the pathogenesis of neurodegenerative diseases [5, 12, 34]. An important challenge in this context is to understand how neurons can tolerate the accumulation of DNA damage and what is the threshold in DNA lesions that trigger neurodegeneration. Neurons are particularly vulnerable to DNA damage because their high metabolic rate which generates ROS. Moreover, it is believed that neurons have a decreased ratio of anti-oxidant to pro-oxidant enzymes that may potentially result in a state of elevated oxidative stress and DNA damage [35]. Importantly, recent studies demonstrate that the high transcriptional activity in a subset of genes that govern crucial neuronal functions, such as early-response genes, can trigger the formation of DSBs [34, 36]. The generation of DSBs is mediated by the activity of topoisomerase IIβ, an enzyme which is robustly expressed in neurons and linked to transcription-related functions [37]. The transcription activity-induced DSBs in a physiological context raises the question of whether the accumulation of unrepaired or erroneously repaired DSBs could result in the formation of PDDF, and potentially contribute to the pathogenesis of neurological diseases [36]. In support of this notion, we have found PDDF in approximately 5 % of non-irradiated SGNs. In this vein, the normal production of DSBs under physiological conditions is exacerbated by the neuronal accumulation of β-amyloid in a murine model of Alzheimer disease [34].

In the case of SGNs, increased vulnerability to DNA damage occurs due to the absence of blood brain barrier in peripheral ganglia that facilitates the access of genotoxic agents, including drugs used in cancer chemotherapy [38–40]. Indeed, DNA damage in SGN seems to be an important component in the long-term peripheral neurotoxicity of anticancer chemotherapy [15, 41].

An important aspect of our results is the neuronal asymmetry in the kinetics of DDR. Indeed, PDDF appear in approximately 30 % of the global population of SGNs at 15 days post-IR while the majority of neurons lack PDDF, indicating effective DNA repair. Different lines of evidence suggest that the kinetics of DDR is related to the individual pattern of transcriptional activity. For example, active neurons break and repair their DNA more often than their less active, “resting”, neighbours, resulting in broad differences in accumulated DNA lesions which will become progressively apparent in the long term [5].

Concerning the structural organization of PDDF reported here, it is well-known in non-neuronal cultured cell lines that DDR occurs in the chromatin landscape. Thus, the DDR requires local modifications in chromatin structure that first become more decondensed and accessible to DNA repair factors, followed by restoration of chromatin organization upon completion of DNA repair [1, 13, 42, 43]. Essential factors for these chromatin modifications are posttranslational modifications of histones, including phosphorylation of the H2AX and ubiquitylation of H2A and H2B, as well as chromatin chaperones and ATP-dependent remodeling factors [44]. Our electron microscopy findings in SGNs allowed us to characterize chromatin modifications in neuronal PDDF. Important hallmarks of PDDF are the super-relaxation of chromatin, which appears as cleared nuclear areas, the sharp boundary between damaged chromatin and the adjacent euchromatin, and the complete transcriptional silencing. The existence of cleared nuclear areas was initially reported in the classical study of Cavanagh et al. [38] in rat SGNs treated with adriamycin, a genotoxic drug used in chemotherapy, although the authors did not relate this finding with DNA damage. Interestingly, the large-scale chromatin decompaction in PDDF occurs in an euchromatic environment, while heterochromatin clumps lack immunolabeling for DNA damage and repair factors. This indicates the higher vulnerability to DNA damage of the euchromatin, which is transcriptionally active or poised for activation [45]. The fine structure of PDDF is characterized by a loose network of fibers ranging from 30 nm chromatin fibers [46] to 11 nm. This finding supports the notion that unfolded 30 nm fiber intermediates provide a structural scaffold which is accessible for signaling and repair factors, as the selective and strong 53BP1 immunogold labeling of the fibers reflects. Therefore, the relaxed state of chromatin in PDDF fits the requirement for DNA repair process, which takes place on exposed DNA [47]. Furthermore, two of the factors enriched in neuronal PDDF, WRAP53 and Ub-H2A, have been implicated in DNA repair in cellular models of DNA damage. Mechanistically, WRAP53 targets the ubiquitin E3 ligase RNF8 to DSBs where ubiquitylated histones H2A and H2AX promote the assembly of repair factor such as 53BP1 [22, 24].

The sharp ultrastructural boundary between the damaged chromatin of PDDF and adjacent euchromatin supports that unrepaired genes congregate and isolate in these damage/repair foci to reduce genome instability and preserve global transcription in undamaged euchromatin. We hypothesized that the sequestration of DNA lesions in one or two individual PDDF allows the neuron to tolerate the accumulation of unrepaired DNA without triggering apoptotic pathways. The molecular barriers between damaged and undamaged chromatin in PDDF are unknown. Interestingly, a recent molecular study, using a new cell-based DSB inducible system to characterize the chromatin landscape around DSB, demonstrates that the recruitment of cohesin prevents γH2AX spreading [48]. The authors propose that, in addition to the function in chromatin architecture, cohesin helps to isolate active genes from damaged ones carrying DSBs. The compartmentalization of neuronal DNA damage might involve DSB-containing chromosome domains moving over relatively large distances to be clustered in PDDF. This interpretation is consistent with previous observations in cell lines revealing increased mobility of the break sites upon IR treatment [49, 50]. Accordingly, in yeast and mammalian cell lines exposed to IR, the formation of DSBs in the nucleolus and heterochromatin results in the movement and relocalization of breaks to the periphery of these nuclear structures [51, 52].

Our results provide the first demonstration of a complete transcriptional silencing at DNA damage foci in neurons. Three lines of evidence support gene silencing within PDDF: i) the total absence of 5’-FU incorporation, a precursor for RNA synthesis, demonstrated with the high resolution of the immunogold electron microscopy, ii) the absence of immunogold labeling for the RNA polymerase II phosphorylated on Ser 2, a well established marker of the elongation phase of transcription [29, 53], and iii) the lack of concentration of RNAs, as indicated the preferential cytochemical staining for ribonucleoproteins [25]. Moreover, transcriptional repression corresponds exactly to the local distribution of γH2AX, which spreads on the entire PDDF but does not propagate on active genes in adjacent euchromatin. Therefore, the expression of γH2AX can be used as a reliable marker of gene silencing in DNA damaged neurons. Transcriptional silencing at PDDF can be essential to reduce genome instability by preventing the synthesis of aberrant mRNA and protein products encoded by damaged genes. In accordance with the PDDF enrichment in p-ATM and Ub-H2A, recent molecular studies have reported an ATM-dependent transcriptional repression at DSBs mediated by H2A ubiquitylation, whereas deubiquitylation of H2A restores transcription [23].

An intriguing aspect of PDDF is their preferential spatial association with the nucleolar and less frequently, nuclear periphery. Interestingly, both nuclear domains are enriched in constitutive heterochromatin, characterized by the abundance of repeated DNA sequences and the binding of HP1 proteins, and correlates with transcriptional silencing [54, 55]. Positioning of PDDF at these repressive nuclear environments may facilitate transcriptional silencing of damaged genes and contribute to maintain genomic stability within PDDF. Alternatively, but not mutually exclusive, nucleolus- and heterochromatin-associated PDDF may represent repair centers for damaged ribosomal genes and repeated DNA sequences of heterochromatin, respectively. In this vein, in non-neuronal culture cell lines exposed to IR, the relocation of DSBs have been reported from the initial induction site in the nucleolus or heterochromatin to the periphery of both structures [51, 52, 56, 57]. Further ChIP-seq analysis of the γH2AX-binding DNA will be necessary to determine what genes are enriched in neuronal PDDF, and particularly if they contain damaged ribosomal genes and repeated sequences of heterochromatin.

## Conclusion

In conclusion, our results strongly indicate that unrepaired DNA in neurons is sequestrated in one to three discrete PDDF of transcriptionally silent chromatin. This transcriptional silencing can be essential to preserve genome stability and prevent the synthesis of aberrant mRNA and protein products encoded by damaged genes. Moreover, the expression of γH2AX can be used as a reliable marker of gene silencing in DNA damaged neurons.

### Ethical approval

All procedures were approved by the Bioethical Committee of the University of Cantabria, and were carried out according to the directives of the Council of the European Communities and current Spanish legislation.
